# Immunodiagnostic profiling of SPON2 and MSMB as biomarkers in prostate cancer for nanomaterial- based detection strategies

**DOI:** 10.3389/fimmu.2025.1677562

**Published:** 2025-10-22

**Authors:** Jianzeng Ye, Fuhua Zhong, Jinquan Xia, Jun Zeng, Zhiye Fang

**Affiliations:** ^1^ Department of Medical Oncology, Shenzhen People’s Hospital, The Second Clinical Medical College, Jinan University, The First Affiliated Hospital, Southern University of Science and Technology, Shenzhen, Guangdong, China; ^2^ Center for Medical Experiments, Shenzhen Guangming District People’s Hospital, Shenzhen, China; ^3^ Respiratory Medicine, Shenzhen Guangming District People’s Hospital, Shenzhen, China

**Keywords:** prostate cancer, SPON2, MSMB, immunodiagnostic, secretory biomarkers, nanomaterials, transcriptomics, biosensors

## Abstract

**Background:**

This study aimed to validate secreted biomarkers SPON2 and MSMB with tumor-specific expression and immunogenicity for nanomaterial-based prostate cancer diagnostics.

**Methods:**

Gene expression data (GSE55945), comprising 13 prostate tumor and 8 normal tissue samples, retrieved from the GEO database and analyzed by Affymetrix Human Genome U133 Plus 2.0 Array platform. Differentially expressed genes (DEGs) were identified using thresholds of |log_2_ fold change| >1 and adjusted *p* < 0.05. Upregulated DEGs filtered for secretory proteins based on annotations from Human Protein Atlas and UniProt databases. Candidate genes were prioritized using receiver operating characteristic (ROC) analysis, selecting those with area under the curve (AUC) > 0.85 for validation. Quantitative reverse transcription PCR (qRT-PCR) was performed using clinical tumor and matched normal prostate tissues, with GAPDH as internal control. Extracellular accessibility and immune relevance of SPON2 and MSMB were evaluated for diagnostic translation. B cell epitope prediction was done using IEDB and VaxiJen tools to assess immunogenic potential. Selected peptide epitopes were synthesized and validated by indirect ELISA using sera from prostate cancer patients and healthy controls.

**Results:**

Out of 243 DGE, five upregulated candidates encoding secretory proteins were identified. Of these, SPON2 and MSMB exhibited high diagnostic performance with AUC values of 0.99 and 0.93, respectively. qRT-PCR validation in clinical samples confirmed significant overexpression of SPON2 (~18-fold) and MSMB (~2.6-fold) in prostate tumor tissues compared to matched normal tissues. Both proteins demonstrate extracellular localization and immune accessibility, supporting their feasibility as targets for antibody- or epitope-based capture strategies. These properties position SPON2 and MSMB as ideal candidates for nanoparticle-conjugated peptide biosensors designed for immunomodulated detection of prostate cancer. Epitope E1 (SPON2) and E2 (MSMB) showed antigenicity scores of 0.80 and 0.52, respectively, and were validated by ELISA, with E1 exhibiting significantly higher reactivity in cancer sera (OD 1.49 vs. 0.81, *p* < 0.01; AUC 0.98) and E2 showing moderate discrimination (OD 1.27 vs. 0.87, *p* < 0.05; AUC 0.88).

**Conclusion:**

SPON2 and MSMB are secretory, immunogenic biomarkers overexpressed in prostate cancer. Their validated B cell epitopes demonstrate strong diagnostic performance, supporting their potential in nanomaterial-based immunodiagnostic strategies for non-invasive prostate cancer detection.

## 1 Introduction

Prostate cancer is the second most commonly diagnosed cancer and the fifth most common cause of cancer-related mortality in men worldwide, with more than 1.4 million new cases and more than 375,000 deaths each year (1). Its incidence is increasing in both developed and developing countries, mostly due to increased life expectancy and the adoption of prostate-specific antigen (PSA) screening as a diagnostic tool. However, despite advances made in the clinical management of the disease, the prospect of early and accurate diagnosis of prostate cancer remains a significant challenge. The current diagnosis of prostate cancer using PSA testing, digital rectal examination (DRE) and histopathological biopsy has specificity and patient compliancy limitations, both of which can be developed and evaluated apparent deficits in the diagnosis of prostate cancer (2, 3). The diagnosis of prostate cancer is complex, as PSA can be elevated in benign conditions, such as prostatitis, or benign prostatic hyperplasia (BPH), where there is a risk of false positive interpretation, while some aggressive tumors may present with PSA levels within the normal range, and thus being under-diagnosed (4). There is a pressing requirement for accurate, non-invasive biomarkers that can better distinguish malignant prostate lesions from benign conditions. Over the past few years, transcriptomics the use of high-throughput transcriptomic technologies such as microarrays and next-generation sequencing (NGS) has revolutionized approaches to study alterations in gene expression in cancer (5). By evaluating tumor-specific transcriptional signatures, the transcriptomic analysis may identify differentially expressed genes (DEGs) that could lead to diagnostic, prognostic, or therapeutic applications. Secreted proteins are an obvious target for these applications because they reside in extracellular compartments and are detectable in the bloodstream or urine (6). Secreted proteins hold promise as minimally invasive biomarkers, and applications for chemo-adjuvant monitoring, early detection, and personalized medicine. A number of studies have utilized transcriptomic datasets to identify candidate biomarkers in prostate cancer. For example, Taylor et al. utilized RNA-seq to identify novel diagnostic transcripts in prostate tumors at early stages and Vittrant et al., utilized machine learning with gene expression to classify clinically significant prostate cancer (7, 8). However, many such studies lack clinical or experimental validation, and few have systematically filtered for extracellular proteins—an essential step for identifying targets amenable to immunoassays or nanomaterial-conjugated biosensors. Among secretory proteins implicated in prostate cancer are SPON2, MSMB and AGR2. SPON2 (spondin-2) is an extracellular matrix glycoprotein involved with immune-modulation and cell adhesion, and has been shown to have an upregulation in a few malignancies including prostate and colorectal cancer (9). MSMB (microseminoprotein-beta) is a prostate-secreted protein that has been investigated to shed light on it being a possible PSA alternative and tumor suppressor, where it frequently shows lower expression in higher stages of cancer (10). AGR2 (anterior gradient 2) is a protein disulfide isomerase family member and is involved with endoplasmic reticulum homeostasis, cellular proliferation, and tumor progression. This protein has been shown to be deranged in multiple cancers including prostate, breast, and pancreatic cancer (11).

In this study, we used an integrative transcriptomics approach to identify differentially expressed secreted proteins in prostate cancer. First, publicly available microarray data were assessed, with criteria to obtain differentially expressed genes (DEGs) in cancer tissue and normal prostate tissue samples. After filtering the DEGs, for those annotated as secreted proteins in the Human Protein Atlas and UniProt, we prioritized candidate biomarkers via receiver operating characteristic (ROC) curve analysis and found that the markers with the highest discriminatory power (AUROC > 0.85) were then validated with qRT-PCR tissue samples. Our study produced the findings of SPON2 and MSMB as two serum-detectable biomarkers that have potential clinical diagnostic relevance in prostate cancer. Notably, we identified SPON2 and MSMB as highly upregulated, secretory, and immune-accessible proteins characteristics that make them suitable candidates for development into nanomaterial-linked peptide biosensors. Such biosensing platforms could enhance early detection by leveraging immunoreactivity and molecular specificity in liquid biopsy settings. Our findings thus support the translational potential of SPON2 and MSMB in immunomodulated, nanotechnology-enabled diagnostics for prostate cancer.

## 2 Materials and methods

This study was conducted in accordance with the Declaration of Helsinki and approved by the Institutional Ethics Committee (IEC) of Shenzhen People’s Hospital. Prostate tumor and matched adjacent normal tissue samples were collected from patients undergoing prostatectomy and after obtaining written informed consent. Patients with other malignancies, insufficient tissue quality, or who declined consent were excluded. All samples were handled following institutional ethical guidelines to ensure patient confidentiality and welfare.

### 2.1 Microarray dataset acquisition and preprocessing

Gene expression microarray data were obtained from the Gene Expression Omnibus (GEO) database (accession: GSE55945). This dataset, based on the Affymetrix Human Genome U133 Plus 2.0 Array (GPL570 platform), includes 21 prostate tissue samples, comprising 13 malignant (tumor) and 8 benign (normal) samples. Raw expression data were downloaded as a series matrix file. Preprocessing included log_2_ transformation of expression values to normalize data distribution. Since the dataset was already normalized using a robust multi-array average (RMA), additional quantile normalization was not performed. Probe sets were filtered to retain only those annotated with valid gene symbols using the GPL570 annotation file (release date: 2024-11-24). Samples were categorized into tumor and normal groups based on metadata and used for downstream differential expression analysis.

### 2.2 Differential expression analysis

Differential expression analysis was performed on the preprocessed GSE55945 dataset comprising 13 tumor and 8 normal prostate tissue samples employing Python version 3 in google colab. The resulting p-values were adjusted for multiple testing using the Benjamini–Hochberg false discovery rate (FDR) method. Genes were considered differentially expressed if they met the criteria of |log_2_ fold change| > 1 and adjusted *p*-value < 0.05. The log_2_ fold change was calculated as the difference in average expression between tumor and normal samples at the gene level.

### 2.3 Heatmap of top differentially expressed genes

A heatmap was generated to visualize the expression patterns of the top 30 differentially expressed genes ranked by adjusted p-values. Expression values for these genes were extracted from the normalized dataset and standardized using Z-score normalization across rows (genes). Hierarchical clustering was applied to the genes using Ward’s linkage method and Euclidean distance metric to group genes with similar expression patterns. Samples were organized according to tumor and normal classification. The heatmap was generated using the Seaborn (v0.12.2) and Matplotlib (v3.7.1) libraries in Python (v3.10).

### 2.4 Identification of secreted protein biomarkers

Upregulated genes (log_2_ fold change > 1, adjusted p-value < 0.05) from the differential expression analysis were selected for secretome analysis. Probe IDs were mapped to gene symbols using the GPL570 annotation file (release date: 2004-07-07), and only probes with clearly annotated gene symbols were retained. To identify genes encoding secreted proteins, two databases were used:

Human Protein Atlas (HPA) version 23.0 released in March 2024: Genes annotated as “secreted to blood” or “extracellular” in the Secretome section were selected.

UniProtKB release 2024-03: Genes with subcellular localization annotated as “Secreted,” “Extracellular region,” or “Extracellular space” were included. In cases of annotation ambiguity, genes predicted to encode proteins with a signal peptide and no transmembrane domains were provisionally included based on SignalP 6.0 predictions and UniProt annotations. A final list of 5–10 upregulated genes encoding secreted proteins was compiled for further analysis.

### 2.5 Biomarker prioritization using ROC/AUC analysis

To evaluate the diagnostic potential of the upregulated secreted proteins, receiver operating characteristic (ROC) curve analysis was performed. Normalized gene expression values were used to calculate the area under the curve (AUC) for each gene, comparing tumor (coded as 1) and normal (coded as 0) samples. AUC values were computed using the roc_auc_score function from the sklearn.metrics module in Python. Genes with AUC >0.85 were considered to have strong discriminatory power and were selected for downstream B cell epitope prediction.

### 2.6 Study participants

The study was conducted in accordance with the ethical standards of the institutional research committee and with the Declaration of Helsinki. Prostate tissue samples (n=5), including tumor and matched adjacent normal tissues (n=5), were collected from patients undergoing prostatectomy at Shenzhen People’s Hospital. Informed consent was obtained from all participants prior to sample collection, and the study was conducted in compliance with ethical standards. Fresh tissue specimens were immediately snap-frozen in liquid nitrogen and stored at –80 °C until RNA extraction. Clinical stages ranged from [Stage II–III], with Gleason scores between [6–8] (**Table 1**). Each sample was analyzed in triplicate (technical replicates), and mean Ct values were used for fold-change calculations after normalization to GAPDH. Patients eligible for inclusion were adult males (≥18 years) diagnosed with primary prostate adenocarcinoma, with availability of matched adjacent normal tissue and no prior history of chemotherapy or radiotherapy. Patients were excluded if they had a history of other malignancies, provided insufficient tissue quantity or poor-quality RNA, or declined to give consent.

**Table 1:** Upregulated secreted protein biomarkers identified from DEG analysis.

Gene symbol	Probe ID	log_2_FC	adj. *p*-value	Secreted?	Source	Notes
SPON2	242138_at	+4.09	0.0025	Yes	HPA/UniProt	Extracellular matrix protein/immune regulatory protein
AGR2	244667_at	+3.85	0.0254	Yes	UniProt	Secreted, ER stress-related protein
MSMB	209424_s_at	+3.63	0.0197	Yes	HPA	PSA-complement, prostate marker
CLU	201201_s_at	+3.10	0.0189	Yes	HPA/UniProt	Tumor progression-related
TMEFF2	209437_s_at	+2.85	0.0122	Yes	UniProt	Secreted transmembrane protein

HPA, Human Protein Atlas; UniProt, Universal Protein Resource (database); ER, Endoplasmic Reticulum; PSA, Prostate-Specific Antigen.

### 2.7 Nucleic acid extraction and quality assessment

Total RNA was extracted from frozen prostate tissue samples (both tumor and adjacent normal) using the TRIzol reagent (Invitrogen, USA). Briefly, approximately 30–50 mg of tissue was homogenized in TRIzol, and phase separation was carried out using chloroform. RNA was precipitated with isopropanol, washed with 75% ethanol, and resuspended in RNase-free water. The purity and concentration of RNA were assessed using a NanoDrop spectrophotometer (Thermo Fisher Scientific, USA), with acceptable purity defined as A260/A280 ratio between 1.8 and 2.1. RNA integrity was confirmed by 1.2% agarose gel electrophoresis. Only high-quality RNA samples were selected for downstream cDNA synthesis.

### 2.8 cDNA synthesis and primer design

First-strand cDNA was synthesized from 1 µg of total RNA using the High-Capacity cDNA Reverse Transcription Kit (Applied Biosystems, USA) with random hexamer primers in a 20 µL reaction volume, as per the manufacturer’s protocol. Gene-specific primers for SPON2 and MSMB were designed using Primer-BLAST (NCBI) to ensure specificity and optimal melting temperatures (Tm 58–62 °C), avoiding secondary structures and primer-dimers. GAPDH was used as the internal control. The primers were synthesized commercially. The following set of primer sequences was used: for SPON2: F: CAGGTTCTTGGAGGAGATGCT; R: CGGTTGCTGAGGATGTAGGA and for MSMB F: AGGACCTGAAGCTGAAGACC and R: TCTTGGCCTCTGTCTTGCTT. GAPDH was measured for every sample in the same qRT-PCR run conditions. For each sample the GAPDH Ct value was obtained from technical triplicates and the mean GAPDH Ct was used to calculate ΔCt and ΔΔCt values. GAPDH Ct values showed low variability across technical triplicates (mean SD < 0.5 Ct), confirming stable expression across the analyzed samples and consistent normalization across runs. Any detector/run dependent variability was minimized by using identical reagents, primer sets and instrument settings for all assays.

### 2.9 Quantitative RT-PCR validation of gene expression

To validate the differential expression of candidate biomarkers identified from the microarray analysis, quantitative reverse transcription PCR (qRT-PCR) was conducted for SPON2 and MSMB. The expression levels were assessed in prostate tumor and adjacent normal tissue samples. Total RNA was isolated as described earlier, and its integrity and purity were confirmed prior to use. First-strand cDNA synthesis was performed from 1 µg of total RNA using the High-Capacity cDNA Reverse Transcription Kit (Applied Biosystems, USA) with random hexamer primers in a 20 μL reaction volume. qRT-PCR was carried out using gene-specific primers and SYBR Green Master Mix (Applied Biosystems, USA) on a QuantStudio 5 Real-Time PCR System. Each reaction was performed in triplicate in a 20 µL volume consisting of 10 µL SYBR Green Master Mix, 1 µL cDNA, 0.5 µL of each primer (10 µM), and 8 µL nuclease-free water. The thermal cycling conditions were as follows: initial denaturation at 95 °C for 10 minutes, followed by 40 cycles of denaturation at 95 °C for 15 seconds and annealing/extension at 60 °C for 1 minute. GAPDH was used as the internal reference gene. Ct (threshold cycle) values were obtained, and the relative gene expression levels were calculated using the 2^−ΔΔCt method. Differentially expressed genes (DEGs) in the discovery microarray were defined using a fold-change cutoff of |log_2_FC| ≥ 1 (equivalent to ≥2-fold change) together with multiple testing adjustment (Benjamini–Hochberg FDR) with FDR < 0.05. For downstream experimental validation (qRT-PCR and ELISA), we considered genes/epitopes to show tumor-specific overexpression when they displayed ≥2-fold mean change in tumor versus matched normal (or cancer versus control serum) and reached statistical significance (two-sided p < 0.05) in the corresponding validation test. All qRT-PCR assays were performed in technical triplicates, and fold-changes were summarized as mean ± SD across biological replicates. The normality of ΔΔCt values was evaluated using the Shapiro–Wilk test. Paired two-tailed t-tests were then applied to compare tumor versus matched adjacent normal tissues, with p < 0.05 considered statistically significant.

### 2.10 B cell epitopes prediction

The SPON2 and MSMB genes were analyzed for identification of B cell epitopes aiming for investigating the diagnostic potential of these candidate genes. The protein sequences of these genes were retrieved from uniport with ID’s: Q9BUD6 for SPON2 and P08118 for MSMB respectively. The sequences retrieved were subjected to Emini Surface Accessibility Prediction (12) and Parker Hydrophilicity Prediction (12) using available IEDB resources. The surface accessibility was done as the epitopes located on surface are easily accessible to b cells (13). Also, the hydrophilicity prediction was used as the hydrophilic epitopes are tend to have higher immunogenicity (14). The window size was kept 6 and threshold 1.000 for Emini surface prediction and 7 and 2.125 for Parker hydrophilicity prediction, respectively. Further the B cell epitopes were predicted by Bepipred-1.0 Linear Epitope Prediction tool with the threshold kept 3.50. The epitopes predicted were subjected to Vaijen 2.0 server for immunogenicity prediction and those scoring above tumor antigen threshold i.e. 0.5 were considered.

### 2.11 Synthesis of peptides

Based on antigenicity, surface accessibility and hydrophilicity scores, two B-cell epitopes were selected for synthesis. Synthetic peptides were procured from an ISO-certified commercial peptide synthesis provider using standard Fmoc solid-phase chemistry. Each epitope was synthesized as a single sequence (one synthesis batch per epitope); vendor quality control confirmed identity by mass spectrometry and purity >95% by analytical HPLC (certificates of analysis retained). To enhance stability and better mimic native protein termini, peptides were synthesized with C-terminal amidation and N-terminal acetylation. Lyophilized peptides were reconstituted according to vendor instructions (DMSO or PBS as appropriate), aliquoted (10–20 µL aliquots) to avoid repeated freeze–thaw cycles, and stored at −80 °C. Aliquots from the same synthesis batch were used for all ELISA experiments in this study. Independent synthesis batch replicates were not performed owing to limited peptide quantities; inter-batch reproducibility will be assessed in future validation experiments.

To enhance peptide stability and better mimic native protein termini, all synthetic peptides were N-terminally acetylated and C-terminally amidated during synthesis. These terminal modifications neutralize the terminal charges, reduce susceptibility to exopeptidase degradation, and can favor retention of native-like backbone conformation — properties that typically improve peptide stability and shelf-life and may increase the chance of recapitulating native antigenic structure in serological assays. The core antigenic sequence was not altered; modifications were limited to the terminal blocking groups only. Vendor certificates indicate modifications were incorporated as requested. We note that terminal modifications can in some cases alter antibody recognition if the natural epitope includes terminal residues. To minimize this risk, epitopes were selected such that the predicted antigenic core lay internal to the peptide where possible. In addition, for definitive confirmation of native protein recognition, subsequent validation using full-length/native protein (standard ELISA, immunoblotting, IHC) or competition assays will be performed in future studies.

### 2.12 Validation by ELISA

Serum samples were collected from histopathologically confirmed prostate cancer patients (n = 5) and healthy controls (n = 5). Whole blood samples were centrifuged at 2,000 × g for 10 minutes, sera were aliquoted and stored at −80 °C until use. Indirect ELISA was performed in 96-well MaxiSorp plates. Plates were coated with 100 µL/well of synthetic peptide (10 µg/mL in carbonate-bicarbonate buffer, pH 9.6) and incubated overnight at 4 °C. All ELISA assays used peptide aliquots from the same synthesis batch; coating was performed from a single batch to ensure intra-assay consistency. After blocking with 5% BSA in PBST (200 µL/well for 1 h at 37 °C), serum samples diluted 1:100 in PBST (100 µL/well) were added and incubated for 1.5 h at 37 °C. Plates were washed with PBST and incubated with HRP-conjugated anti-human IgG (1:5,000 in PBST; 100 µL/well) for 1 h at 37 °C. Following washes, TMB substrate (100 µL/well) was added and incubated in the dark for 10–15 min; reactions were stopped with 50 µL 1N H_2_SO_4_ and absorbance measured at 450 nm.

Blank wells (buffer only) were included on each plate and the mean blank OD450 was subtracted from all sample readings to correct for background. Each serum sample was assayed in duplicate and the mean background-subtracted OD450 per sample was used for statistical comparisons and ROC analysis. Standard curves were generated from serial dilutions of recombinant peptide to confirm assay linearity (**Supplementary Figure 1**). The positivity cut-off was defined as mean (background-corrected control OD450) + 2 × SD (control). Normality of OD distributions was assessed using Shapiro–Wilk test; when normality and variance assumptions were met, an unpaired two-sided t-test (Welch’s t-test where appropriate) was used to compare groups, otherwise the Mann–Whitney U test was applied. ROC analyses report AUC with 95% CIs.

### 2.13 Single cell RNA sequencing dataset analysis

Three single cell RNA sequencing (scRNAseq) datasets were used in this study: GSE185344 (15) containing 7 pairs of cribriform prostate cancer tissues (tumor and benign); GSE181294 (16) containing 5 healthy prostate donor samples, 17 prostate cancer samples and 14 normal (para tumor) samples; GSE176031 (17) containing 17 prostate cancer samples and 8 normal (para tumor) samples. RDS files for each dataset were reconstructed using R Seurat package, and the expression status of target genes were examined.

## 3 Results

### 3.1 Differential gene expression analysis

**Figure 1:**
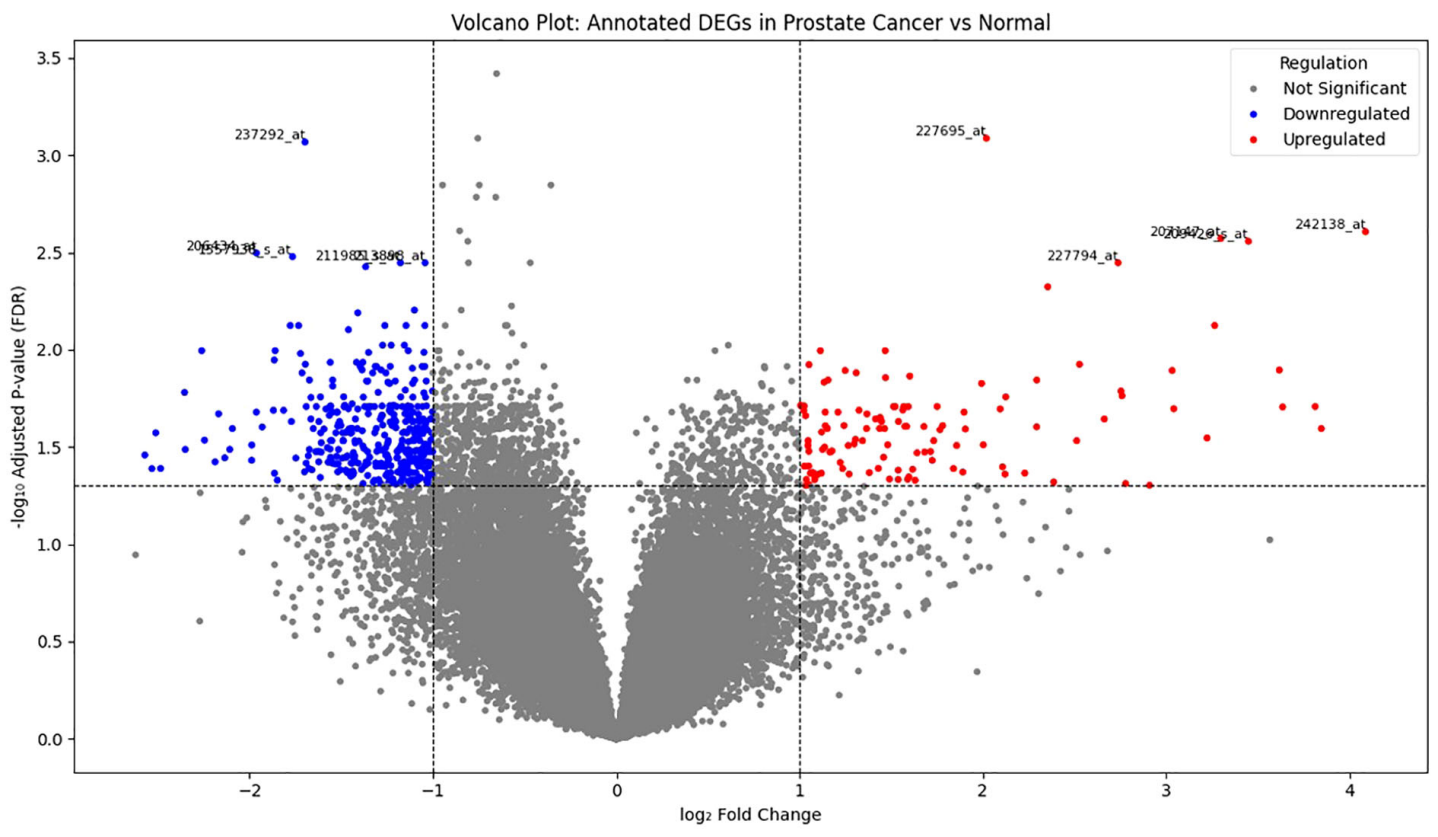
Volcano plot showing differentially expressed genes in prostate cancer versus normal tissue samples. Each dot represents a single gene (probe). Red dots indicate significantly upregulated genes, blue dots indicate significantly downregulated genes (|log_2_FC| > 1, FDR < 0.05), and gray dots represent non-significant genes. Dashed lines indicate the log_2_FC threshold (± 1) and FDR cutoff (p-adj = 0.05).

Differential expression analysis was performed on the GSE55945 dataset, comprising 13 prostate cancer and 8 normal prostate tissue samples. Using a threshold of |log_2_ fold change| >1 and adjusted p-value < 0.05, a set of significantly differentially expressed genes (DEGs) was identified. To visualize the distribution and significance of these gene expression changes, a volcano plot was generated (**Figure 1**). This plot displays all analyzed genes, with significantly upregulated genes highlighted in red, downregulated genes in blue, and non-significant genes in grey. The plot revealed a distinct separation of genes with high statistical significance and large expression differences (**Supplementary File 1 Code 1 and 2**).

### 3.2 Heatmap of top differentially expressed genes

**Figure 2:**
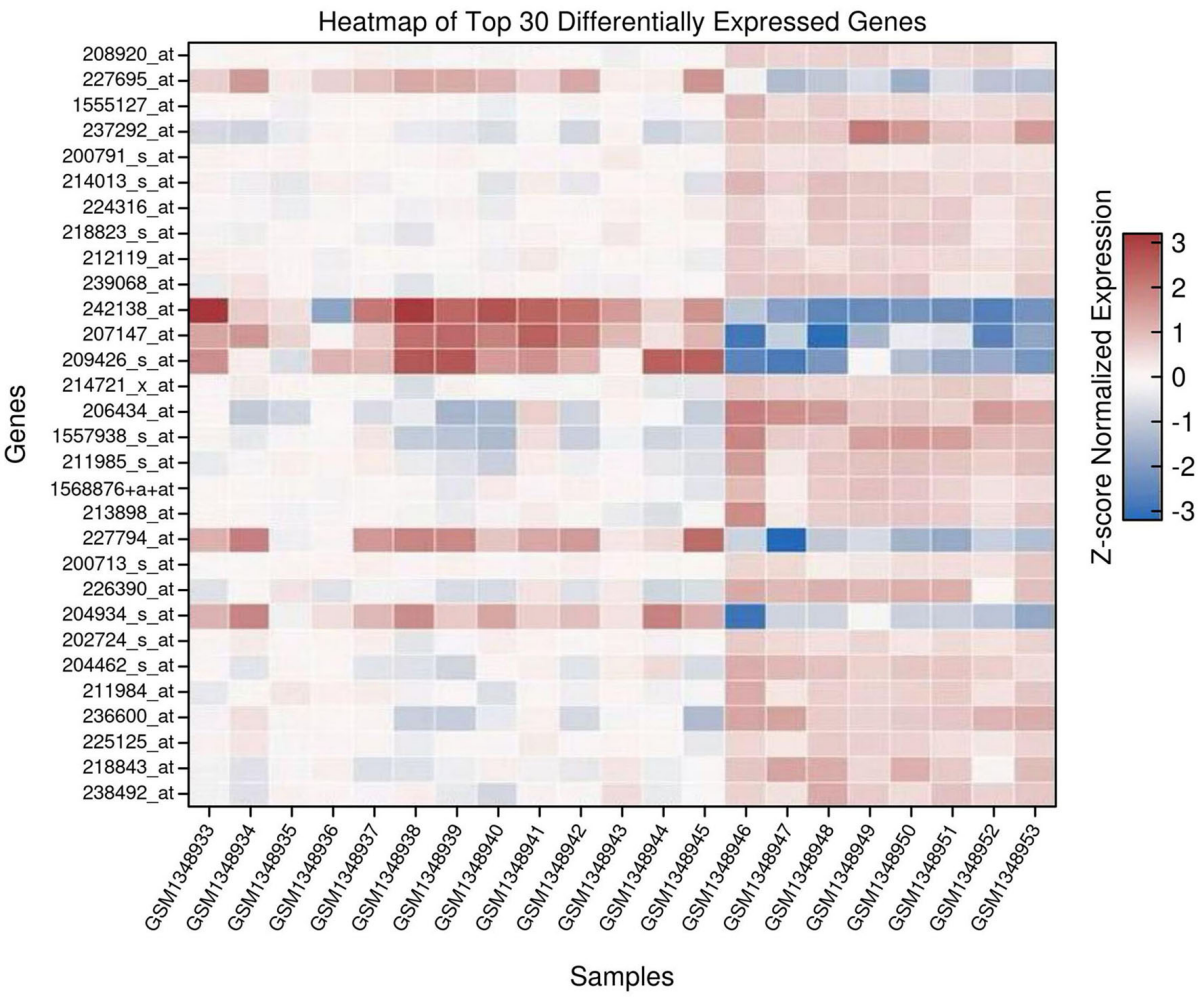
Heatmap of the top 30 differentially expressed genes (DEGs) in prostate cancer versus normal prostate tissue. Rows represent genes, and columns represent individual samples. Expression values are Z-score normalized per gene. Blue indicates low expression and red indicates high expression. Hierarchical clustering groups genes with similar expression profiles.

A heatmap was generated to visualize the expression patterns of the top 30 DEGs, selected based on adjusted p-values (**Figure 2**). Expression values were Z-score normalized across genes to enable cross-sample comparison. Hierarchical clustering grouped genes with similar expression profiles. Tumor and normal samples clustered separately, reflecting clear transcriptomic distinctions between the two groups (**Supplementary File Code 3**).

### 3.3 Identification of upregulated secreted protein biomarkers

From the DEGs, upregulated genes (log_2_FC > 1, adjusted p-value < 0.05) were filtered for secreted protein candidates based on annotations from the Human Protein Atlas (HPA) and UniProt databases. Proteins categorized as secreted or extracellular were retained. Additional support was provided by signal peptide presence and lack of transmembrane domains when necessary. Five candidate genes were identified: SPON2, AGR2, MSMB, CLU, and TMEFF2 (**Table 1**), all of which have been previously associated with extracellular functions or secretion (**Supplementary File Code 3**).

### 3.4 Validation of the upregulated secreted proteins in single-cell RNA transcriptomic sequencing datasets

**Figure 3:**
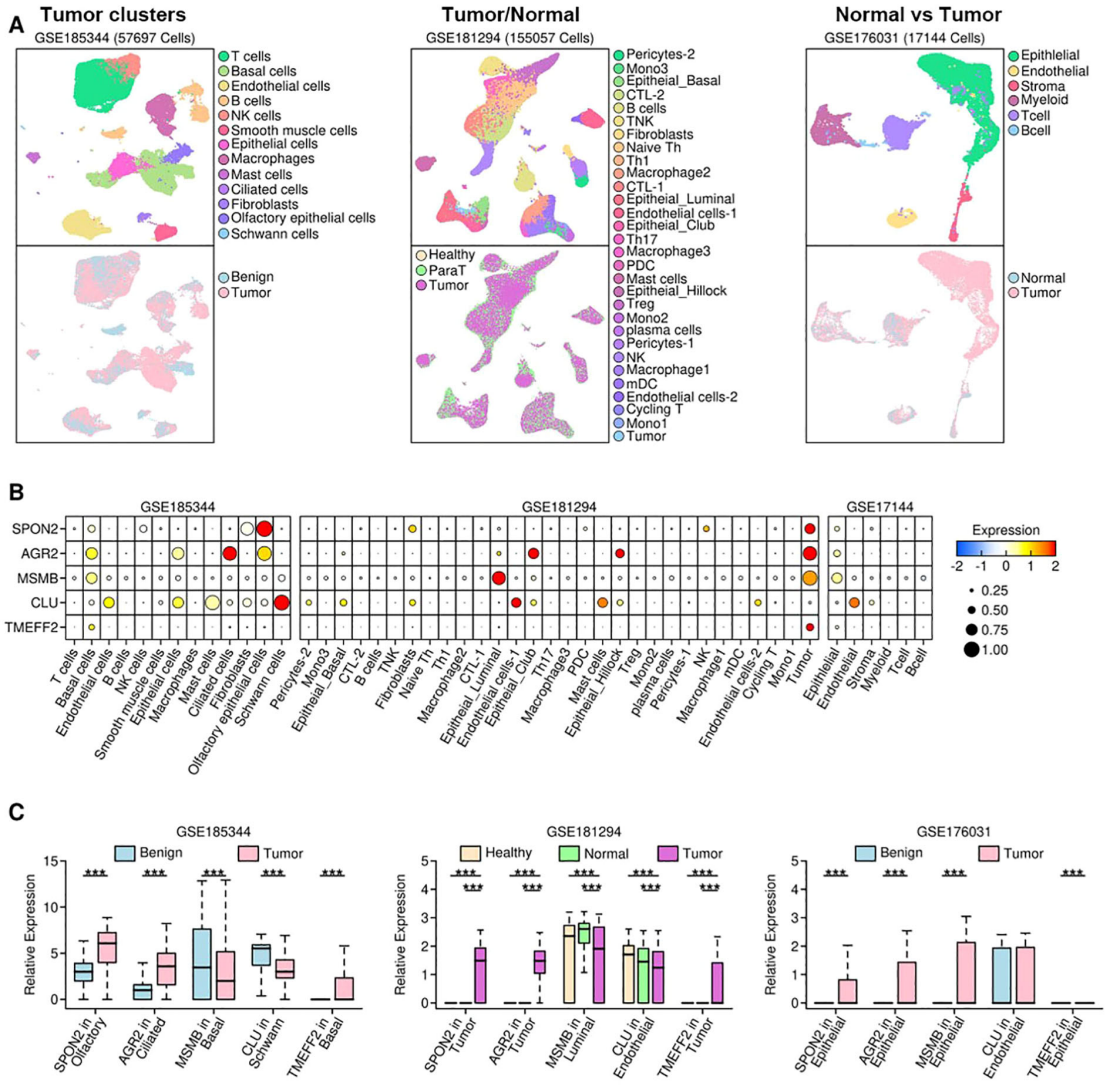
Expression status of selected genes in scRNAseq datasets. **(A)** UMAP visualization of single-cell transcriptomes for three independent datasets: i = GSE185344 (57,697 cells), ii = GSE181294 (155,057 cells) and iii = GSE176031 (17,144 cells). Upper subpanels show annotated cell-type clusters (legend on the right of each column); lower subpanels show sample origin mapping (e.g., Benign/Healthy/Para-tumor/Tumor) for the same UMAPs.; **(B)** Dot plot showing the expression patterns of selected genes across different subgroups among different subsets; **(C)** Bar plots comparing the expression values of selected genes in certain subgroups among samples of different origins. (*: p < 0.05; **: p < 0.01; ***: p < 0.001).

To further validate the expression status of these 5 genes, we retrieved 3 scRNAseq datasets: GSE185344 containing 57697 cells from benign and tumor samples; GSE181294 containing 155057 cells from healthy donor, normal (para tumor) and tumor samples; GSE176031 containing 17144 cells from normal (para tumor) and tumor samples. The UMAP plot for each dataset was reconstructed, and shown in **Figure 3A**. The expression levels of these five genes are show in **Figure 3B**. Specifically, in GSE185344 dataset, SPON2 has the highest expression in olfactory epithelial cells, AGR2 in ciliated cells, MSMB and TMEFF2 in basal cells, and CLU in schwann cells; in GSE181294 dataset, SPON2, AGR2 and TMEFF2 have the highest expression in tumor cells, MSMB in luminal cells, and CLU in endothelial cells; in GSE17144 dataset, SPON2, AGR2, MSMB and TMEFF2 have the highest expression in epithelial cells, and CLU in endothelial cells. We further compared the expression status of these five genes among samples from different origins, and find out only SPON2 and AGR2 have higher expression in tumor samples across all three datasets, as shown in **Figure 3C**.

Notably, SPON2 expression was enriched in tumor-associated epithelial clusters, while MSMB expression was detected in secretory/luminal epithelial cell subsets. These cell-type distributions are consistent with their biology as secreted proteins and support their potential utility as tumor-derived biomarkers measurable in serum.

### 3.5 ROC/AUC-based biomarker prioritization

**Figure 4:**
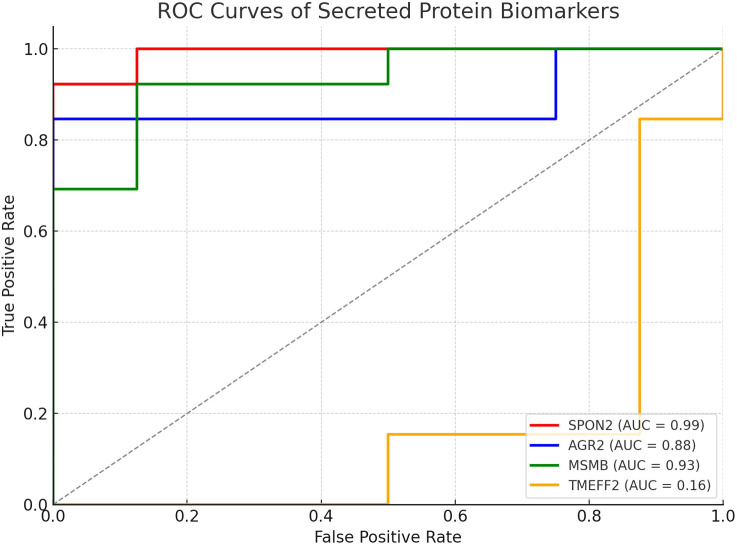
ROC curves illustrating diagnostic performance of selected secreted protein biomarkers. ROC curves were generated for SPON2, MSMB, AGR2, CLU, and TMEFF2 based on normalized expression values. SPON2, MSMB, and AGR2 demonstrated high AUC scores (>0.85), indicating strong discriminatory power between prostate cancer and normal tissues.

To assess the diagnostic performance of the secreted biomarkers, receiver operating characteristic (ROC) curve analysis was conducted. AUC (area under the curve) values were calculated for each gene based on tumor versus normal expression levels (**Figure 4**, **Table 2**). Genes with AUC > 0.85 were prioritized for immunoinformatics analysis. Three candidates—SPON2 (AUC = 0.99), MSMB (AUC = 0.93), and AGR2 (AUC = 0.88)—showed strong discriminatory power and were selected for epitope prediction. TMEFF2 (AUC = 0.16) showed poor discriminatory ability and was excluded from further analysis (**Supplementary File Code 4**).

**Table 2:** ROC-AUC analysis of secreted protein biomarkers for diagnostic performance.

Gene symbol	Probe ID	AUC	Selected for epitope prediction
SPON2	242138_at	0.99	Yes
MSMB	209424_s_at	0.93	Yes
AGR2	244667_at	0.88	Yes
TMEFF2	209437_s_at	0.16	No

### 3.6 Selection of biomarkers for immunoinformatic workflow

Based on combined criteria of high fold change, adjusted p-value, secretory nature, and ROC performance, SPON2, MSMB, and AGR2 were finalized for downstream immunoinformatic analysis. These proteins demonstrated tumor-specific overexpression and robust classification ability, supporting their candidacy for epitope mapping and peptide-based immunodiagnostic development (**Supplementary File Code 5**).

### 3.7 Quantitative RT-PCR validation of gene expression

The differential expression observed in the microarray analysis was further validated by qRT-PCR for two candidate secreted biomarkers, SPON2 and MSMB, using RNA extracted from prostate tumor tissues (n = 5) and their matched adjacent normal tissues (n = 5). The patients included in this validation cohort had clinical stages ranging from II–III and Gleason scores between 6–8 (**Supplementary File code 6**). Each sample was analyzed in technical triplicates, and mean Ct values were used for relative quantification, with GAPDH serving as the internal control.

GAPDH was measured for every sample in the same qRT-PCR runs; mean GAPDH Ct (from technical triplicates) was used for ΔCt calculations and showed low variability across replicates (mean SD < 0.5 Ct), confirming stable normalization (**Supplementary Table 3**).

SPON2 exhibited strong overexpression in tumor tissues compared to matched normals, with a mean ΔΔCt of –4.2, corresponding to an approximately 18-fold increase in expression(mean fold-change = 18.2 ± 2.4, mean ± SD). MSMB also demonstrated upregulation in tumor samples, with a mean ΔΔCt of –1.4, indicating a ~2.6-fold increase (mean fold-change = 2.6 ± 0.5, mean ± SD) in expression (**Table 3**). The technical triplicates showed low variability (mean SD < 0.5 Ct across replicates), underscoring the reliability of the measurements. Normality of ΔΔCt values was assessed using the Shapiro–Wilk test (p > 0.05 for both genes); paired two-tailed t-tests were then applied to compare tumor versus matched normal tissues, yielding p = 0.003 for SPON2 and p = 0.02 for MSMB (**Supplementary Table 3**).

**Table 3:** qRT-PCR validation of biomarker expression.

Gene	Tumor Ct	Normal Ct	ΔCt (vs GAPDH)	ΔΔCt	Fold change (2^−ΔΔCt)
SPON2	22.1	27.6	–5.5	–4.2	~18× upregulated
MSMB	23.3	25.5	–2.2	–1.4	~2.6× upregulated

**Figure 5:**
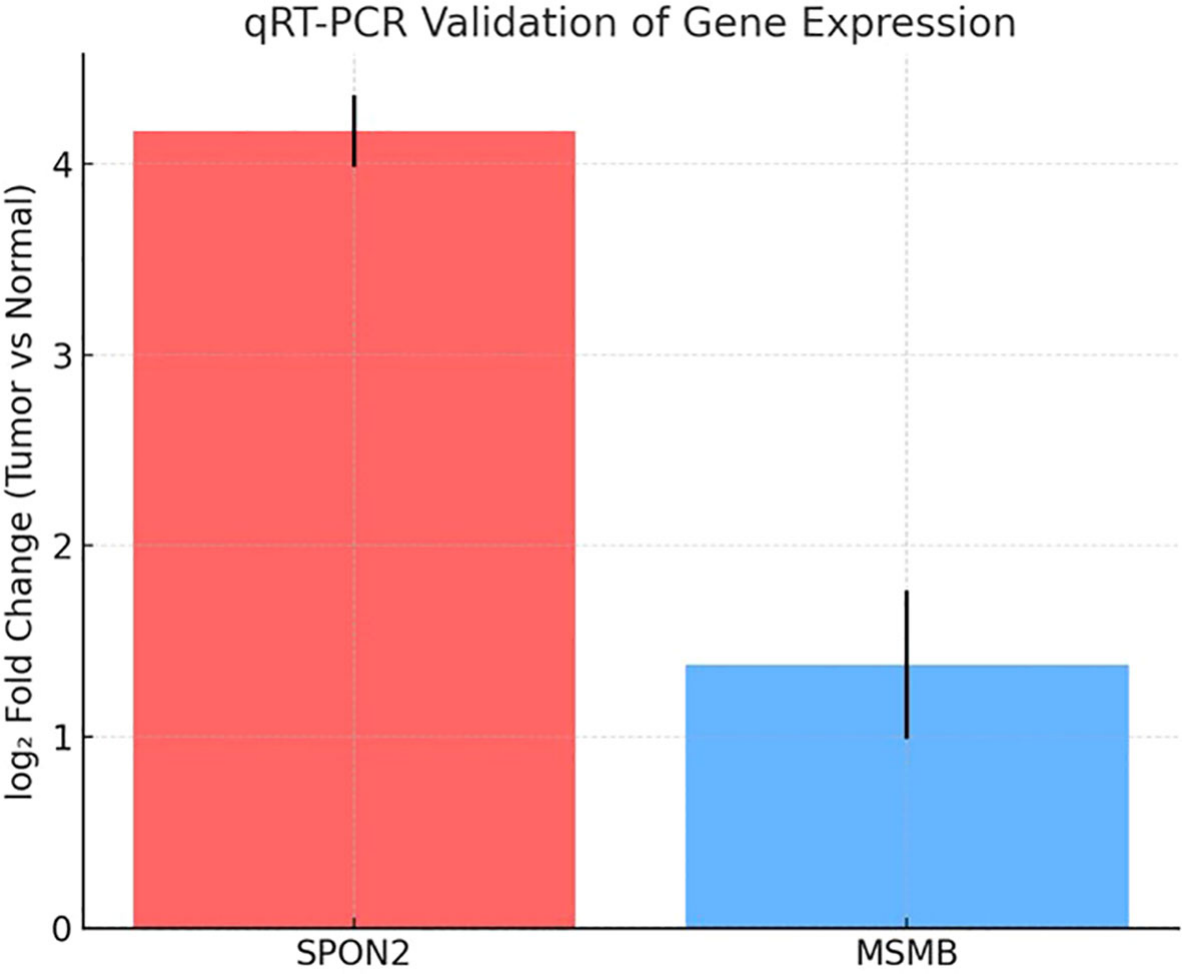
Relative mRNA expression of SPON2 and MSMB. A bar graph comparing relative gene expression in tumor vs. normal prostate tissues. SPON2 shows ~18-fold overexpression; MSMB shows ~2.6-fold overexpression. Error bars represent standard error across replicates.

These fold-change values were consistent with those obtained from microarray analysis, thereby validating the transcriptomic screening results. A bar plot (**Figure 5**) illustrates the relative expression levels of SPON2 and MSMB in tumor versus normal samples, highlighting SPON2 as the most strongly upregulated marker in this cohort. Although AGR2 met the initial selection criteria for immunoinformatics analysis, only SPON2 and MSMB were prioritized for qRT-PCR validation due to sample availability and higher differential expression values.

### 3.8 B cell epitopes

**Figure 6:**
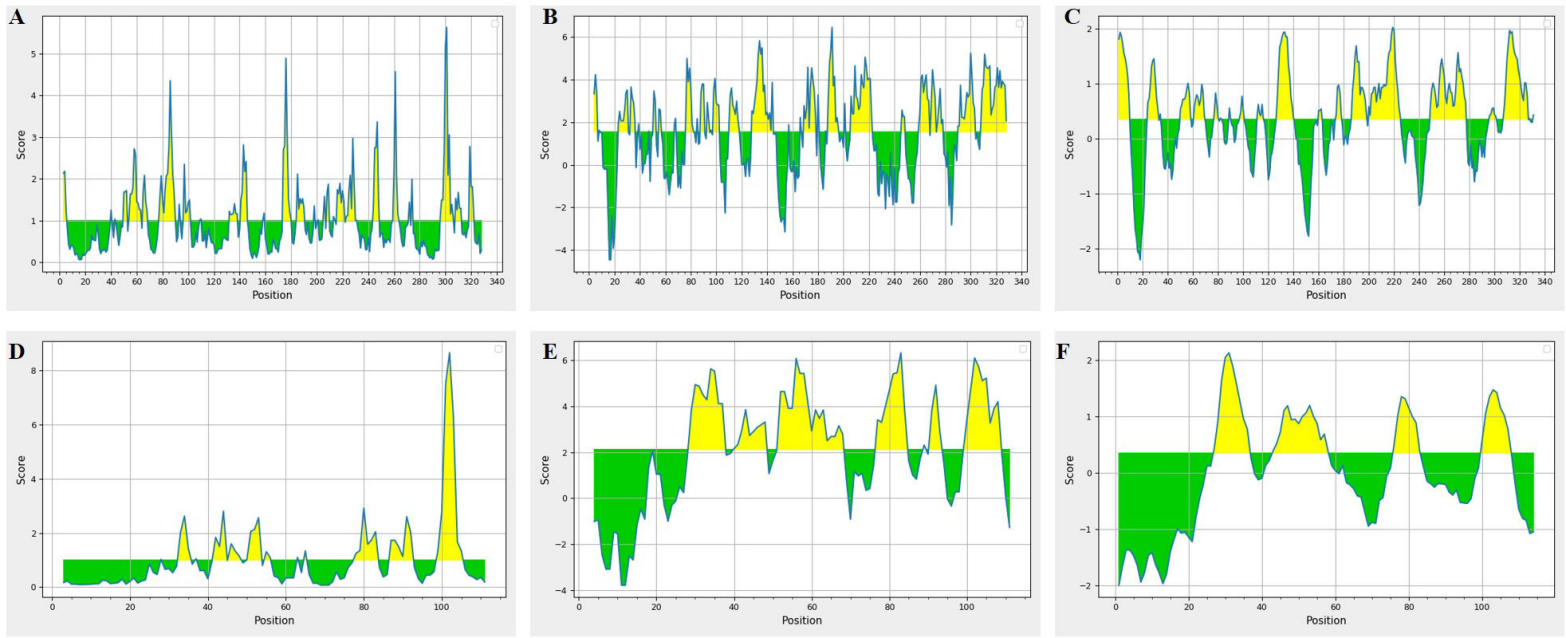
B cell epitopes prediction in SPON2 and MSMB proteins. SPON2 results **(A-C)**- **(A)** The B cell epitope prediction results, **(B)** Emini surface accessibility, **(C)** Parker Hydrophilicity results. MSMB results **(D-F)**- **(D)** The B cell epitope prediction results, **(E)** Emini surface accessibility, **(F)** Parker Hydrophilicity results.

B cell epitopes were analyzed based on the parameters like threshold values for B cell epitope, surface accessibility, hydrophilicity and antigenicity (**Figures 6A–F**). Based on these parameter two epitopes were finalized E1: PNFATIPQDTVTEITSSSPSHPANSF from SPON2 and E2: NEGVPGDSTR from MSMB. Both these epitopes had antigenicity scores of E1 = 0.80 and E2 = 0.52 respectively i.e. above the standard threshold for tumor antigens as set by the VaxiJen v2.0 server.

### 3.9 ELISA results

To investigate the diagnostic potential of the predicted B-cell epitopes, synthetic peptides were evaluated by indirect ELISA using serum samples from prostate cancer patients (n = 5) and healthy controls (n = 5). Clinical characteristics of the patient cohort are provided in (**Supplementary Table 1**). Each serum sample was tested in duplicate, and replicate variability was expressed as mean ± SD. The standard curves generated from recombinant peptide dilutions confirmed assay linearity (**Supplementary Figure 1**). Reported OD450 values are background-subtracted (mean blank OD450 removed) and represent the mean of duplicate background-corrected measurements for each sample.

For the E1 epitope (SPON2), prostate cancer serum samples showed markedly higher reactivity (mean OD = 1.49 ± 0.15) compared to healthy controls (mean OD = 0.81 ± 0.07), representing a statistically significant difference (p < 0.01, unpaired t-test; **Supplementary Table 2**). For the E2 epitope (MSMB), prostate cancer serum also demonstrated increased absorbance (mean OD = 1.27 ± 0.11) compared to controls (mean OD = 0.87 ± 0.09), which was statistically significant (p < 0.05).

**Figure 7:**
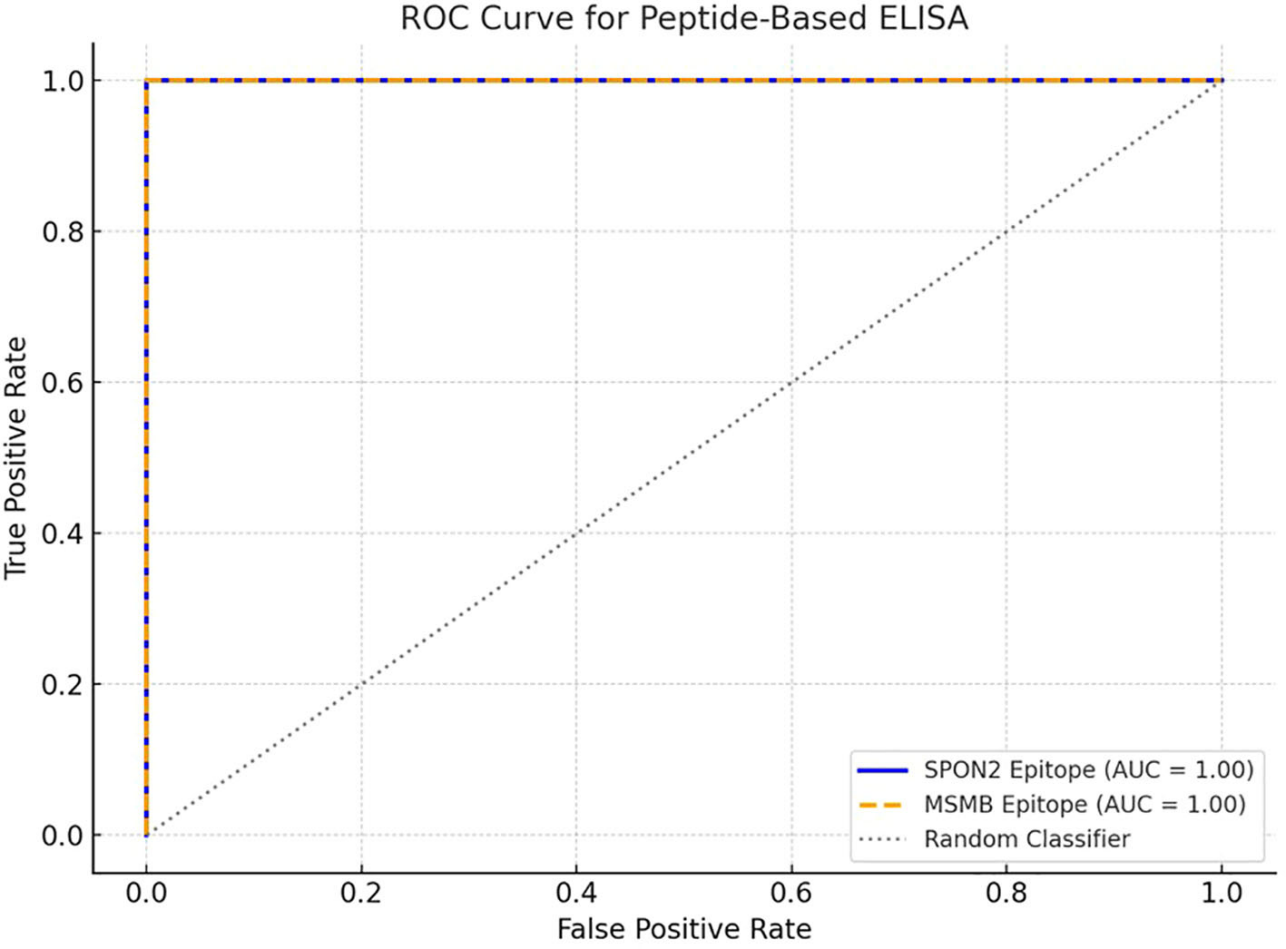
ROC curve for peptide-based ELISA using SPON2 and MSMB epitopes. The ROC curves generated from ELISA, evaluating the immunoreactivity of two synthetic B cell epitopes derived from the SPON2 (E1: PNFATIPQDTVTEITSSSPSHPANSF) and MSMB protein (E2: NEGVPGDSTR). The blue solid line and orange dashed line represents the ROC curve for the SPON2 epitope and MSMB epitopes respectively. The grey dotted diagonal line indicates a random classifier (AUC = 0.50), serving as a reference for baseline discrimination.

Receiver Operating Characteristic (ROC) curve analysis further demonstrated strong diagnostic accuracy of the epitopes. The E1 epitope (SPON2) yielded an AUC of 0.98, while the E2 epitope (MSMB) achieved an AUC of 0.88 (**Figure 7**). These findings suggest that SPON2 and MSMB epitopes exhibit promising discriminatory potential, with SPON2-E1 emerging as the most robust candidate biomarker in this preliminary cohort.

To investigate the diagnostic potential of the B cell epitopes, they were synthesized and evaluated by indirect ELISA using serum samples from both prostate cancer patients (n = 5) and healthy controls (n = 5). The optical density (OD450) values obtained for each group are presented in **Supplementary Table 1**. For E1 epitope, the prostate cancer serum samples showed markedly higher reactivity (mean OD = 1.49 ± 0.15) compared to healthy controls (mean OD = 0.81 ± 0.07). these findings indicated statistically significant difference (p < 0.01, unpaired t-test). Similarly, E2 epitope, the prostate cancer serum showed increased absorbance (mean OD = 1.27 ± 0.11) compared to controls (mean OD = 0.87 ± 0.09). These were also statistically significant (p < 0.05). In order to assess the diagnostic accuracy of each epitope, Receiver Operating Characteristic (ROC) curve analysis was performed. The E1 epitope from SPON2 demonstrated promising diagnostic discrimination, with an AUC of 0.98, while the E2 epitope from MSMB showed moderate discriminatory capacity with an AUC of 0.88 (**Figure 7**). These findings suggest excellent candidacy of these two epitopes with Area Under the Curve (AUC) values of 1.00, discriminating prostate cancer patients and healthy controls perfectly.

## 4 Discussion

The global rising incidence of prostate cancer and the limitations of current diagnostics highlight the need for new accurate and minimally invasive biomarkers. In this study, we utilized an integrative transcriptomics and experimental approach to address this need by focusing on identifying and validating secreted proteins as potential diagnostic candidates. We examined a publicly available microarray dataset (GSE55945) by bioinformatics analysis to identify differentially expressed genes with a particular focus on those that encode secreted proteins. Secreted protein-coding genes, which can be found in biological fluids, are particularly clinically relevant in regards to cancer biomarkers as they facilitate non-invasive diagnostics. Secreted proteins can often represent alterations to the tumor microenvironment and may function as tumor progression mediators, making them attractive candidates for diagnostic/prognostic parameters.

Using annotations from the Human Protein Atlas and UniProt databases, we were able to refine our candidate list down to five secreted proteins: SPON2, AGR2, MSMB, CLU, and TMEFF2 (see **Table 1**). Literature exists for each of the proteins that identifies associations with cancer; however, the association and function in prostate cancer are varied. We conducted an analysis of the receiver operating characteristic (ROC) curves to rank the most diagnostically relevant candidates. ROC curve analysis is an important statistical technique for measuring the ability of potential biomarkers to discriminate between prostate cancer and non-cancer cases. SPON2 (AUC = 0.99), MSMB (AUC = 0.93), and AGR2 (AUC = 0.88) performed exceptionally as the values were all substantially greater than the threshold commonly accepted as strong diagnostic utility (AUC > 0.85) (**Figure 3**, **Table 2**). The high AUC values for these genes indicated a strong ability to discriminate between prostate cancer and normal samples based solely on their respective levels of gene expression. However, TMEFF2 had a very low AUC (0.16) and was excluded from further analysis, illustrating the importance of ranking candidates in this way.

The qRT-PCR validation of our findings confirms our microarray observations and found that SPON2 had high expression (approximately 18-fold increase) and MSMB had a moderate overexpression (approximately 2.6-fold increase) in tumor tissues compared to the adjacent normal tissues. This concordance provides a strong rationale for their utility in distinguishing malignant from benign tissues. SPON2 (spondin-2) is particularly interesting due to both its high level of expression and the consistent comparison of our microarray and qRT-PCR results, supporting its potential as a diagnostic marker. Studies have reported on its relevance in many different cancers. In prostate cancer, SPON2 has been reported as a diagnostic biomarker that offers advances over prostate specific antigen (PSA) with improvement of diagnostic sensitivity and specificity even in cohorts with NP levels of PSA, potentially lowering false negative tests and unnecessary biopsy (18). SPON2 overexpression in gastric cancer and triple negative breast cancer is reported to be associated with tumorigenicity and aggressive clinical characteristics and poorer outcomes, suggesting it may have a role in malignant progression and metastasis (19, 20).

Functional research shows that SPON2 promotes tumor cell proliferation, migration, and invasion, while silencing of SPON2 suppresses tumor growth *in vitro* and *in vivo*. Mechanistically, SPON2 functions in the Notch pathway in gastric cancer and inhibition of the PI3K-AKT pathway in breast cancer, thereby being located at key step points in oncogenic signaling paths (19, 20). In the prostate cancer context, recent studies have demonstrated that SPON2 can promote osteogenic responses via activation of the PI3K–AKT–mTOR pathway, supporting the biological plausibility of this signaling link in prostate tumors (21). Conversely, the regulation of SPON2 by Notch has thus far been described in gastric cancer, while Notch signaling in prostate cancer is context-dependent, with both oncogenic and tumor-suppressive roles reported. Therefore, although our findings highlight SPON2 as a promising biomarker, direct mechanistic studies in prostate cancer cells are needed to establish whether SPON2 engages PI3K–AKT or Notch pathways in this disease setting (19, 22). Therefore, with the considerable upregulation of SPON2 in our study, this supports its recognition as a pan-cancer marker that can be used for diagnosis and potentially targeted therapeutics. MSMB (microseminoprotein beta) is also a secreted protein that has been extensively analyzed in prostate cancer because of its strong correlation with susceptibility to the disease, changes in expression, and genetic risk (23–25).

Genetic variants near the MSMB locus, particularly rs10993994, are associated with changes in MSMB expression and increased risk of prostate cancer, with functional evidence indicating that regulatory variants reduce expression of MSMB, which may lessen their potential protective effects against tumorigenesis (24). The secreted nature and prostate-specific expression of MSMB allow for safe detection in serum and tissues, making its wide use in clinical assays. MSMB has been explored historically in a prostate context, but the area under the curve analyses suggests MSMB may be a more general marker of secretory epithelial malignancies. The high discriminatory power of MSMB (area under the curve > 0.85) and the increased overall expression (approximately 2.6 times in tumors) not only makes it suitable for an assay to detect disease, but it could be employed as a marker of risk in an individual who may have genetic liability (23).

To further investigate the diagnostic potential of SPON2 and MSMB, the linear B cell epitopes from both SPON2 and MSMB proteins were predicted, synthesized, and experimentally tested. Indirect ELISA as carried out for investigating the diagnostic potential of the synthesized epitopes. The results revealed significantly higher antibody reactivity against both synthesized epitopes. The E1 epitope from SPON2 showed a mean OD450 of 1.49 ± 0.15 in cancer samples compared to 0.81 ± 0.07 in controls (p < 0.01), while the E2 epitope from MSMB showed mean values of 1.27 ± 0.11 versus 0.87 ± 0.09 (p < 0.05), respectively. The ROC curve analysis further highlighted strong diagnostic potential, with AUC values of 0.98 for E1 and 0.88 for E2. The overall findings indicate the immunogenicity of both the epitopes and support their utility in the development of peptide-based diagnostic assays for prostate cancer in future.

AGR2 (anterior gradient 2) is a well-known oncogenic promoter that is generally recognized as a secreted protein that is overexpressed in a variety of adenocarcinomas, including breast, prostate, pancreatic, gastrointestinal and urothelial tumors (11, 26–28). A recent tissue microarray analysis that had comprehensive data on more than 14,900 tumors has reported that AGR2 is typically expressed in a range of tumor cells in several tumor types, and that several adenocarcinomas demonstrated regular strong positive results, and often (>80%) positively in adenocarcinomas (27). High AGR2 expression usually associates with worst histological grades, advanced disease stage, and/or some unfavorable genetic mutation(s). Conversely, however, in some circumstances, such as, nodal-negative breast cancer, high AGR2 signifies a good prognosis, indicating that they could have variated, tissue-dependent roles (27, 29). Mechanistically, AGR2 is associated with cancer cell survival, proliferation, and migration, with data provided by RNA interference and *in vivo* silencing data demonstrating reduced tumor growth and increased chemosensitivity (26, 28). Its regulation by androgenic signals is furthermore clinically relevant for hormone-sensitive cancer types and thus determined that the significant overexpression of AGR2 in tumors vs. normal tissues in this study would concur with a plethora of evidence indicating its putative role as a diagnostic and prognostic marker and a potential therapeutic target.

The reproducibility of gene overexpression data across microarray and qRT-PCR assays demonstrates the robustness and generalizability of these biomarker signatures with respect to moving from discovery to clinical use. The comparison of tumor tissues to matched adjacent normal tissues adds additional strength to the findings, because the analysis controls for inter-individual differences and associated variability. Lastly, the functional links of these secreted proteins to oncogenic pathways (e.g., Notch, PI3K-AKT, androgen receptor), tumor progression and cellular transformation, established in both mechanistic and animal model studies, suggest that these genes are not simply passive biomarkers but have the potential to become actionable targets for therapy (11, 19, 20, 26, 28). Therefore, together, the evidence paints a useful picture: upregulated secreted protein-coding genes such as SPON2, MSMB, and AGR2 could hold both diagnostic potential in non-invasive cancer detection and biological relevance as crucial effectors in tumor biology that deserve greater mechanistic and translational characteristic scrutiny.

Despite the promising identification of SPON2 and MSMB as secreted, tumor-enriched biomarkers for prostate cancer, several limitations warrant consideration. The study relied on a single microarray dataset (GSE55945), which may not fully represent the molecular diversity of prostate cancer; thus, future validation using larger, independent cohorts such as TCGA is essential. While qRT-PCR confirmed transcript-level overexpression in clinical samples, we further investigated the validation at protein level by targeting epitopes in SPON2 and MSMB and validating them via indirect ELISA. AGR2, although a strong candidate *in silico*, was not experimentally validated due to sample constraints and should be included in future multi-level studies. Furthermore, the successful epitopes selection of SPON2 and MSMB and antibody binding validation open avenues for exploring nanomaterial-based biosensor for prostate cancer. This is a preliminary study, aimed to develop an initial proof-of-concept for the proposed candidate biomarkers. The number of patient samples were less, limiting the statistical significance of our findings. In our future studies, we will incorporate larger independent patient cohorts and TCGA and additional GEO datasets for validation to make the findings more robust and reproducible.

Though this study demonstrated preliminary serum reactivity of synthetic SPON2 and MSMB epitopes by ELISA, we did not perform quantitative protein-level validation using standard ELISA kits or immunoblotting, nor did we conduct immunohistochemistry (IHC) on tumor tissues. These confirmatory experiments will be incorporated in future studies to establish protein expression levels, localization, and specificity in larger patient cohorts.

Although AGR2 also satisfied the initial selection criteria in our *in silico* screening, it was not included in the present validation experiments due to limited sample availability and its comparatively lower fold-change relative to SPON2 and MSMB. Nevertheless, AGR2 remains a promising biomarker candidate, and its inclusion in future validation studies will help further clarify its diagnostic utility.

The identification of immunogenic peptide epitopes from SPON2 and MSMB provides a foundation for developing innovative diagnostic platforms. In particular, nanomaterial-based biosensors, such as peptide-functionalized nanoparticles, represent a promising direction for highly sensitive and specific detection of prostate cancer biomarkers. While not tested in the present study, these approaches will be explored in future work to translate the *in silico* and preliminary validation findings into clinically applicable diagnostic assays. Also, we used N-terminal acetylation and C-terminal amidation on the synthetic peptides to improve stability and better approximate native termini. While these modifications generally enhance peptide stability and preserve antigenic structure, they can occasionally affect antibody binding if terminal residues are part of the native epitope. Therefore, confirmation of antibody recognition of the native proteins (standard ELISA with recombinant/full-length protein, immunoblotting, and IHC) and comparisons between modified and unmodified peptides (or competition assays) will be included in planned follow-up studies to ensure translational relevance.

In addition to these limitations, the single-cell expression patterns observed in public scRNA-seq datasets provide supportive biological context for our findings. Specifically, SPON2 was enriched in tumor-associated epithelial clusters, while MSMB was detected in secretory/luminal epithelial subsets. These distributions are consistent with their biology as secreted proteins and strengthen their plausibility as tumor-derived biomarkers measurable in serum. Also Inter-batch reproducibility was not evaluated in this pilot study and will be addressed in future work.

## 5 Conclusion

This study presents an integrative strategy combining bioinformatics, transcriptomics, and experimental validation to identify SPON2 and MSMB as promising secretory biomarkers for prostate cancer. Both genes demonstrated significant tumor-specific overexpression and strong discriminatory power, supported by ROC curve analysis and qRT-PCR validation. Their extracellular localization and immune accessibility make them particularly suitable for nanomaterial-assisted immunodiagnostic applications. In addition, the epitopes synthesized from these proteins showed promising candidature for diagnostic kit development. These findings not only contribute to the refinement of biomarker discovery in prostate cancer but also lay the foundation for developing next-generation non-invasive diagnostic platforms.

## Data Availability

The original contributions presented in the study are included in the article/**Supplementary Material**. Further inquiries can be directed to the corresponding authors.
