# Supplementary material for: Immunodiagnostic profiling of SPON2 and MSMB as biomarkers in prostate cancer for nanomaterial- based detection strategies
Source: Front Immunol. 2025 Oct 22;16:1677562. doi: 10.3389/fimmu.2025.1677562 (PMC12586104; doi:10.3389/fimmu.2025.1677562)
Supplement: Supplementary file 1 [file DataSheet1.pdf]

Supplementary file 1

Immunodiagnostic Profiling of SPON2 and MSMB as Biomarkers in Prostate Cancer for Nanomaterial-Based Detection Strategies

Code 1 (DEG identification):

```
import platform

import ssl

import urllib.request

annot_url = "https://ftp.ncbi.nlm.nih.gov/geo/platforms/GPL5nnn/GPL570/annot/GPL570.annot.gz"

annot_path = "/mnt/data/GPL570.annot.gz"

ssl._create_default_https_context = ssl._create_unverified_context

urllib.request.urlretrieve(annot_url, annot_path)

annot_df = pd.read_csv(annot_path, sep="\t", comment='#', low_memory=False)

probe_to_gene = annot_df.set_index("ID")["Gene Symbol"]

deg_results["Gene Symbol"] = deg_results.index.map(probe_to_gene)

sig_degs["Gene Symbol"] = sig_degs.index.map(probe_to_gene)

sig_degs = sig_degs.dropna(subset=["Gene Symbol"]).drop_duplicates(subset=["Gene Symbol"])

sig_degs.head(10)


import seaborn as sns

import matplotlib.pyplot as plt

# Categorize DEGs

deg_df["category"] = "Not Significant"

deg_df.loc[(deg_df["adj_pval"] < 0.05) & (deg_df["log2FC"] > 1), "category"] = "Upregulated"

deg_df.loc[(deg_df["adj_pval"] < 0.05) & (deg_df["log2FC"] < -1), "category"] = "Downregulated"

# Plot

plt.figure(figsize=(10, 6), dpi=300)

palette = {'Upregulated': 'red', 'Downregulated': 'blue', 'Not Significant': 'gray'}

sns.scatterplot(data=deg_df, x="log2FC", y=-np.log10(deg_df["adj_pval"]),

                hue="category", palette=palette, s=8, linewidth=0, alpha=0.8)
```

```
# Add lines
plt.axhline(y=-np.log10(0.05), color='black', linestyle='--', linewidth=0.5)
plt.axvline(x=1, color='black', linestyle='--', linewidth=0.5)
plt.axvline(x=-1, color='black', linestyle='--', linewidth=0.5)
plt.xlabel(r'$\log_2$ FC', fontsize=12)
plt.ylabel(r'$-\log_{10}$ FDR', fontsize=12)
plt.title("Volcano Plot: Prostate Cancer vs Normal", fontsize=14)
plt.legend(title="", loc='upper right')
plt.tight_layout()
plt.show()
```

Code 2 Volcano plot:

```
fc_threshold = 1

pval_threshold = 0.05

# Classify DEGs

deg_results["Significance"] = "Not Significant"

deg_results.loc[(deg_results["adj_p_value"] < pval_threshold) & (deg_results["log2FC"] > fc_threshold),
"Significance"] = "Upregulated"

deg_results.loc[(deg_results["adj_p_value"] < pval_threshold) & (deg_results["log2FC"] < -fc_threshold),
"Significance"] = "Downregulated"

# Plot

plt.figure(figsize=(10, 7))

sns.scatterplot(

    data=deg_results,

    x="log2FC",

    y=-np.log10(deg_results["adj_p_value"]),

    hue="Significance",

    palette={"Upregulated": "blue", "Downregulated": "red", "Not Significant": "lightgrey"},

    edgecolor=None,

    s=30

)

# Annotate top up/down genes

top_genes = deg_results[(deg_results["adj_p_value"] < 0.01) & (deg_results["log2FC"].abs() > 2)].copy()

top_genes = top_genes.sort_values(by="adj_p_value").head(10)

for idx, row in top_genes.iterrows():

    plt.text(row["log2FC"], -np.log10(row["adj_p_value"]), idx, fontsize=8)


plt.axvline(x=fc_threshold, color='black', linestyle='--', linewidth=1)

plt.axvline(x=-fc_threshold, color='black', linestyle='--', linewidth=1)

plt.axhline(y=-np.log10(pval_threshold), color='black', linestyle='--', linewidth=1)

plt.title("High-Resolution Volcano Plot (Tumor vs Normal)", fontsize=14)

plt.xlabel("Log2 Fold Change", fontsize=12)

plt.ylabel("-Log10 Adjusted P-value", fontsize=12)
```

```
plt.legend(title="Gene Regulation", loc='upper right')

plt.grid(True, linestyle='--', linewidth=0.5, alpha=0.7)

plt.tight_layout()

plt.show()

# Download and read the GPL570 annotation file (Affymetrix Human Genome U133 Plus 2.0 Array)

!wget -q https://ftp.ncbi.nlm.nih.gov/geo/platforms/GPL5nnn/GPL570/annot/GPL570.annot.gz

import pandas as pd

annot = pd.read_csv("GPL570.annot.gz", sep="\t", skiprows=27)

# Create a mapping dictionary

probe_to_gene = dict(zip(annot["ID"], annot["Gene Symbol"]))


# Map DEGs to gene symbols

deg_df["Gene Symbol"] = deg_df.index.map(probe_to_gene)

deg_df_with_symbols = deg_df.dropna(subset=["Gene Symbol"]).drop_duplicates("Gene Symbol")


# Top upregulated genes with gene names

upregulated = deg_df_with_symbols[(deg_df_with_symbols["log2FC"] > 1) & (deg_df_with_symbols["adj_pval"]
< 0.05)]

upregulated.sort_values("log2FC", ascending=False).head(10)
```

Code 3 (Top 30 genes selection and heat map)

```
# Selecting top 30 significant genes by adjusted p-value
top_gene_ids = deg_df.sort_values("adj_pval").head(30).index
# Subset the expression matrix for those genes
heatmap_data = expression_df.loc[top_gene_ids]
# Z-score normalization (per gene) for better heatmap contrast
heatmap_data = heatmap_data.subtract(heatmap_data.mean(axis=1), axis=0)

# Re-run after session reset: Loading the uploaded GSE55945 microarray dataset
import pandas as pd
import gzip
from io import StringIO
file_path = "/mnt/data/GSE55945_series_matrix.txt.gz"
with gzip.open(file_path, 'rt') as f:
    lines = f.readlines()
# Extract data block from the series matrix
start_idx = lines.index("!series_matrix_table_begin\n") + 1
end_idx = lines.index("!series_matrix_table_end\n")
data_str = ''.join(lines[start_idx:end_idx])
expression_df = pd.read_csv(StringIO(data_str), sep="\t", index_col=0)
# Assign group labels (13 tumor, 8 normal)
group_labels = ['tumor'] * 13 + ['normal'] * 8
expression_df = expression_df.iloc[:, :len(group_labels)]
group_series = pd.Series(group_labels, index=expression_df.columns)
# Log2 transform if needed
import numpy as np
if expression_df.max().max() > 100:
    expression_df = np.log2(expression_df + 1)
# Perform differential expression analysis
from scipy.stats import ttest_ind
from statsmodels.stats.multitest import multipletests
```

```
tumor_samples = group_series[group_series == 'tumor'].index
normal_samples = group_series[group_series == 'normal'].index
t_stats, p_values = ttest_ind(expression_df[tumor_samples], expression_df[normal_samples], axis=1)
adj_pvals = multipletests(p_values, method='fdr_bh')[1]
log2fc = expression_df[tumor_samples].mean(axis=1) - expression_df[normal_samples].mean(axis=1)
deg_df = pd.DataFrame({
    'log2FC': log2fc,
    'adj_pval': adj_pvals
})
# Select top 30 DEGs based on adjusted p-value
top30_genes = deg_df.sort_values("adj_pval").head(30).index
heatmap_data = expression_df.loc[top30_genes]
# Z-score normalization across rows (genes)
heatmap_data_zscore = heatmap_data.subtract(heatmap_data.mean(axis=1), axis=0)
# Plot the heatmap
import seaborn as sns
import matplotlib.pyplot as plt
plt.figure(figsize=(12, 10))
sns.heatmap(heatmap_data_zscore, cmap="vlag", xticklabels=False, yticklabels=True,
            linewidths=0.5, cbar_kws={"label": "Z-score"})
plt.title("Heatmap of Top 30 Differentially Expressed Genes")
plt.xlabel("Samples")
plt.ylabel("Genes")
plt.tight_layout()
plt.show()
```

Code 4 (Secreted proteins):

```
import pandas as pd
import numpy as np
import gzip
from io import StringIO
from sklearn.metrics import roc_auc_score, roc_curve, auc
import matplotlib.pyplot as plt
file_path = "/mnt/data/GSE55945_series_matrix.txt.gz"
with gzip.open(file_path, 'rt') as f:
    lines = f.readlines()
# Extract the expression matrix
start_idx = lines.index("!series_matrix_table_begin\n") + 1
end_idx = lines.index("!series_matrix_table_end\n")
data_str = ''.join(lines[start_idx:end_idx])
expression_df = pd.read_csv(StringIO(data_str), sep="\t", index_col=0)
# Assign group labels
group_labels = ['tumor'] * 13 + ['normal'] * 8
expression_df = expression_df.iloc[:, :len(group_labels)]
group_series = pd.Series(group_labels, index=expression_df.columns)
# Log2 transform
if expression_df.max().max() > 100:
    expression_df = np.log2(expression_df + 1)
# Defining binary labels
y_true = group_series.map({'tumor': 1, 'normal': 0}).values
# Defining secreted gene probe IDs
secreted_probes = {
    "SPON2": "242138_at",
    "AGR2": "244667_at",
    "MSMB": "209424_s_at",
    "CLU": "201201_s_at",
    "TMEFF2": "209437_s_at"
```

auc_df

Code 5 (AUC/ROC curve):

```
import pandas as pd
import numpy as np
import gzip
from io import StringIO
from sklearn.metrics import roc_auc_score, roc_curve, auc
import matplotlib.pyplot as plt

file_path = "/mnt/data/GSE55945_series_matrix.txt.gz"
with gzip.open(file_path, 'rt') as f:
    lines = f.readlines()
# Extracting the expression matrix
start_idx = lines.index("!series_matrix_table_begin\n") + 1
end_idx = lines.index("!series_matrix_table_end\n")
data_str = ''.join(lines[start_idx:end_idx])
expression_df = pd.read_csv(StringIO(data_str), sep="\t", index_col=0)
# Assigning group labels (13 tumor, 8 normal)
group_labels = ['tumor'] * 13 + ['normal'] * 8
expression_df = expression_df.iloc[:, :len(group_labels)]
group_series = pd.Series(group_labels, index=expression_df.columns)
# Log2 transform
if expression_df.max().max() > 100:
    expression_df = np.log2(expression_df + 1)
# Define binary labels
y_true = group_series.map({'tumor': 1, 'normal': 0}).values
# Define secreted gene probe IDs
secreted_probes = {
    "SPON2": "242138_at",
    "AGR2": "244667_at",
    "MSMB": "209424_s_at",
    "CLU": "201201_s_at",
```

```
    "TMEFF2": "209437_s_at"
}
# Compute and plot ROC curves
plt.figure(figsize=(8, 6))
colors = ['red', 'blue', 'green', 'purple', 'orange']
auc_results = []
for i, (gene, probe_id) in enumerate(secreted_probes.items()):
    if probe_id in expression_df.index:
        y_score = expression_df.loc[probe_id].values
        fpr, tpr, _ = roc_curve(y_true, y_score)
        roc_auc = auc(fpr, tpr)
        auc_results.append((gene, probe_id, roc_auc))
        plt.plot(fpr, tpr, color=colors[i], lw=2,
                 label=f'{gene} (AUC = {roc_auc:.2f})')
# Plot baseline
plt.plot([0, 1], [0, 1], color='grey', lw=1, linestyle='--')
plt.xlim([0.0, 1.0])
plt.ylim([0.0, 1.05])
plt.xlabel('False Positive Rate')
plt.ylabel('True Positive Rate')
plt.title('ROC Curves of Secreted Protein Biomarkers')
plt.legend(loc="lower right")
plt.grid(True)
plt.tight_layout()
plt.show()
# Output AUC table
auc_df = pd.DataFrame(auc_results, columns=["Gene Symbol", "Probe ID", "AUC"]).sort_values("AUC",
ascending=False)
auc_df
```

Supplementary table 1: Clinical characteristics of prostate cancer patients included for qRT-PCR and ELISA validation.

Patient ID	Age (years)	Clinical Stage	Gleason Score	Sample Type Used
P1	62	II	6	Tumor + Matched Adjacent Normal, Serum
P2	67	II	7	
P3	70	III	7	
P4	65	III	8	
P5	72	II	6	

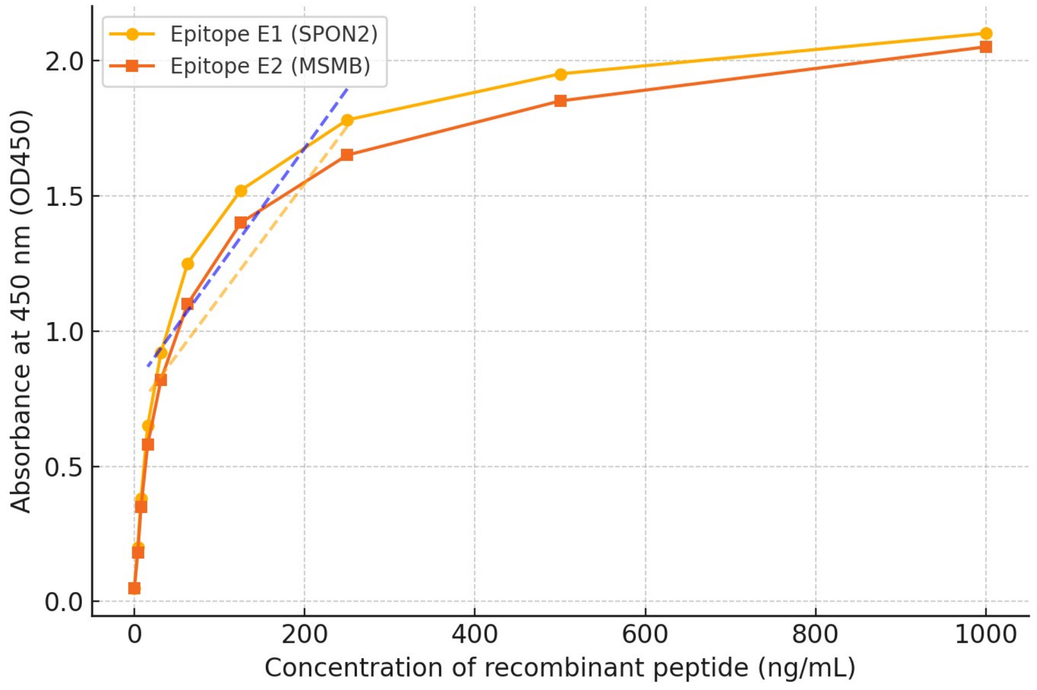

Supplementary figure 1: The standard curves generated from serial dilutions of recombinant peptides (E1 = SPON2, E2 = MSMB).

Supplementary Table 2. ELISA OD450 values for SPON2 (E1) and MSMB (E2) epitope reactivity. Serum samples from prostate cancer patients (n = 5) and healthy male controls (n = 5) were analyzed by indirect ELISA to assess antibody reactivity against synthetic B-cell epitopes derived from SPON2 and MSMB. Higher OD values indicate stronger antigen–antibody interaction. The “Label” column denotes the diagnostic group used for ROC curve analysis, where 1 represents prostate cancer and 0 represents healthy control. These values were used for calculating mean ± SD, statistical comparisons, and ROC-based evaluation of diagnostic performance.

Note: OD450 values shown are background-subtracted (mean blank well OD removed) and are the mean of duplicate measurements.

Group	E1_SPON2_OD	E2_MSMB_OD	Label
Prostate Cancer	1.599343	1.230487	1
Prostate Cancer	1.472347	1.230141	1
Prostate Cancer	1.629538	1.336294	1
Prostate Cancer	1.804606	1.013008	1
Prostate Cancer	1.453169	1.041262	1
**Mean ± SD**	**1.59 ± 0.15**	**1.17 ± 0.13**	—
Healthy Control	0.776586	0.793771	0
Healthy Control	0.957921	0.748717	0
Healthy Control	0.876743	0.881425	0
Healthy Control	0.753053	0.759198	0
Healthy Control	0.854256	0.708770	0
**Mean ± SD**	**0.84 ± 0.07**	**0.78 ± 0.07**	—

Supplementary Table 3. qRT-PCR Ct values, ΔCt, ΔΔCt, and fold-change calculations for SPON2 and MSMB in prostate tumor tissues (n = 5) and matched adjacent normal tissues (n = 5). Note: GAPDH was used as the internal control. Fold-changes were calculated using the 2^−ΔΔCt method. Data illustrate consistent upregulation of SPON2 and MSMB in tumor samples compared to matched normal tissues.

**Patient ID**	**Gene**	**Tumor Ct**	**Normal Ct**	**ΔCt (Tumor-GAPDH)**	**ΔCt (Normal-GAPDH)**	**ΔΔCt (Tumor-Normal)**	**Fold-change (2^ΔΔ-Ct)**
P1	SPON2	24.36	28.26	4.21	8.3	-4.09	16.98
P1	MSMB	24.46	25.91	4.31	5.95	-1.64	3.11
P2	SPON2	23.72	27.81	3.25	7.58	-4.34	20.18
P2	MSMB	24.32	25.81	3.85	5.58	-1.74	3.33
P3	SPON2	23	27.31	2.92	7.88	-4.96	31.1
P3	MSMB	24.26	25.59	4.19	6.17	-1.98	3.96
P4	SPON2	24.34	28.59	4.61	9.01	-4.4	21.06
P4	MSMB	24.34	26.03	4.61	6.45	-1.84	3.57
P5	SPON2	23.41	27.54	3.58	7.51	-3.93	15.22
P5	MSMB	24.3	25.76	4.46	5.73	-1.26	2.4
